# In Peripheral Blood Mononuclear Cells *Helicobacter pylori* Induces the Secretion of Soluble and Exosomal Cytokines Related to Carcinogenesis

**DOI:** 10.3390/ijms23158801

**Published:** 2022-08-08

**Authors:** Josefina Atrisco-Morales, Mónica Ramírez, Carlos Alberto Castañón-Sánchez, Adolfo Román-Román, Ilce Valeria Román-Fernández, Dinorah Nashely Martínez-Carrillo, Samuel García-Arellano, José Francisco Muñoz-Valle, Hugo Alberto Rodríguez-Ruiz, Gloria Fernández-Tilapa

**Affiliations:** 1 Laboratorio de Investigación Clínica, Facultad de Ciencias Químico Biológicas, Universidad Autónoma de Guerrero, Chilpancingo 39087, Guerrero, Mexico; 2 Laboratorio de Investigación en Biomoléculas, Facultad de Ciencias Químico Biológicas, Universidad Autónoma de Guerrero, Chilpancingo 39087, Guerrero, Mexico; 3 CONACYT-Universidad Autónoma de Guerrero, Chilpancingo 39087, Guerrero, Mexico; 4 Laboratorio de Investigación Biomédica, Hospital Regional de Alta Especialidad de Oaxaca, San Bartolo Coyotepec 71256, Oaxaca, Mexico; 5 Laboratorio de Investigación en Bacteriología, Facultad de Ciencias Químico Biológicas, Universidad Autónoma de Guerrero, Chilpancingo 39087, Guerrero, Mexico; 6 Instituto de Investigación en Ciencias Biomédicas, Centro Universitario de Ciencias de la Salud, Universidad de Guadalajara, Guadalajara 44340, Jalisco, Mexico; 7 Laboratorio de Biomedicina Molecular, Facultad de Ciencias Químico-Biológicas, Universidad Autónoma de Guerrero, Chilpancingo 39087, Guerrero, Mexico

**Keywords:** *Helicobacter pylori*, exosomes, cytokines, PBMCs, inflammation, carcinogenesis

## Abstract

*Helicobacter pylori* promotes the secretion of cytokines that regulate inflammation and carcinogenesis. Immune cells secrete cytokines into the extracellular medium or packaged in exosomes. The objective of this study was to analyze the profile of soluble and exosomal cytokines that were secreted by human peripheral blood mononuclear cells (PBMCs) that were infected with *H. pylori* and to build a network of interaction between cytokines and cellular proteins. PBMCs were obtained by density gradient centrifugation and infected with *H. pylori* for 24 h. The infection was verified by immunofluorescence and Western blot for CagA. The exosomes were obtained from culture supernatant by ultracentrifugation and characterized by transmission electron microscopy, particle size analysis, and Western blot for CD9 and CD81. Cytokines were quantified using a multiplex immunoassay in the culture supernatant, intact exosomes, and lysed exosomes. *H. pylori* adheres to lymphocytes and translocates CagA. In PBMCs, *H. pylori* induces an increase in the soluble and exosomal IL-1β, IL-6, TNF-α, IL-10, IL-17A, IL-21, and IL-22. The protein–protein interaction (PPI) network shows that soluble and exosomal cytokines interact with proteins that participate in signaling pathways such as NF-κB, MAPK, PI3K-Akt, Jak-STAT, FoxO, and mTOR, that are related to carcinogenesis; moreover, TNF-α had the highest number of interactions. Cytokine-loaded exosomes represent another means of intercellular communication that is activated by *H. pylori* to stimulate inflammation, carcinogenesis, or cancer progression. Cytokine-loaded exosomes are likely to be associated with extragastrointestinal diseases of inflammatory origin.

## 1. Introduction

*Helicobacter pylori* (*H. pylori*) is a Gram-negative bacterium that colonizes the human stomach, and is the main risk factor for gastritis, peptic ulcer disease, and gastric cancer development [1]. *H. pylori* induces gastric inflammation and is characterized by secretion of the cytokines interleukin (IL)-1β, IL-6, tumor necrosis factor alpha (TNF-α), interferon gamma (IFN-γ), IL-17A, IL-17F, IL-21, IL-22, IL-23, IL-25, IL-4, IL-10, IL-31, IL-33, produced by macrophages, neutrophils, dendritic cells, epithelial cells, and CD4+ T lymphocytes [2]. Various components of the bacterium activate the inflammatory response and adaptive immunity, including the Type 4 secretion system (T4SS), neutrophil-activating protein (HP-NAP), vacuolating cytotoxin A (VacA), cytotoxin-associated gene A (CagA), and the outer membrane proteins (Omp-18) and *H. pylori* adhesin A (HpaA) [3]. In addition, the cytokines IFN-γ, IL-6, and IL-17 promote gastric inflammation and lead to the development of gastric cancer [4]. It has been shown that IL-1β, IL-33, IL-23, and TNF-α increase migration, invasion, and epithelial-mesenchymal transition (EMT) in gastric cancer cell lines AGS, MKN-45, MGC-803, BGC-823, and SGC-7091, cellular processes that are related to tumor progression [5,6,7,8].

Exosomes are small extracellular vesicles (EVs), 30 to 150 nm in diameter, that are involved in local and systemic intercellular communication [9]. The content of exosomes reflects the state of the producing cell since these vesicles can transport various types of molecules such as growth factors, lipids, proteins, DNA, mRNA, microRNAs (miRNAs), long noncoding RNAs (lncRNAs), and cytokines [10]. Cytokines are soluble factors that can also reach the extracellular space packaged or anchored to the surface of exosomes [11]. Cytokines have been detected in exosomes that were recovered from synovial fluid of patients with osteoarthritis (IL-1β, IL-2, IL-4, IL-5, IL-6, IL-13, IL-17, TNF-α, and IFN-γ), plasma from alcohol and cigarette-consuming human immunodeficiency virus (HIV)-positive patients (IL-8 and IL-10), in EVs that were recovered from diabetes mellitus patients (TNF-α), as well as in culture medium of HIV-infected macrophages that were stimulated with cigarette components (IL-6) [12,13,14,15]. In a study that was conducted on patients aged 26–45 years and 65–86 years with gastric cancer, it was found that in exosomes that were isolated from serum, the concentration of TNF-α and transforming growth factor-beta (TGF-β) increases, and that of IL-10 decreases, compared to individuals without cancer. The results that were derived from this study suggest that exosomal cytokines are related to gastric cancer, but not to age [16]. In vitro studies show that in the GES-1 cell line of non-tumor gastric epithelial cells, *H. pylori* induces the secretion of exosomes containing the CagA oncoprotein and that, in the gastric cancer cell lines, AGS and SGC-7901, the bacterium promotes the release of exosomes containing epithelial-mesenchymal factor (MET) [17,18,19]. In the mouse macrophage cell line RAW264.7, *H. pylori* induced the secretion of miR-155-rich exosomes. On the other hand, uninfected RAW264.7 macrophages that were treated with exosomal miR-155 expressed high levels of TNF-α, IL-6, and IL-23 and decreased myeloid differentiation primary response 88 (Myd88) and Nuclear Factor kappa B (NF-κB). These data suggest that exosomal miR-155 is involved in the regulation of the gastric inflammatory response [20].

In line with the above, in GES-1 cells, *H. pylori* promoted the secretion of exosomes with a high content of miR-25, which stimulated the activation of NF-κB and a high expression of IL-6, monocyte chemoattractant protein-1 (MCP-1), vascular cell adhesion molecule 1 (VCAM-1), and intercellular adhesion molecule 1 (ICAM-1) in HUVECs cell line of endothelial cells. These results are explained by the inhibition of Kruppel-like factor 2 (KLF2), the target gene of miR-25, and suggest that exosomal miR-25 contributes to the endothelial dysfunction and the inflammation [21]. The studies that are described, show that *H. pylori* induces the secretion of exosomes in non-cancerous gastric epithelial cells, in cancer cell lines, and in mouse macrophages. In vivo, *H. pylori*-infected human gastric mucosa is infiltrated by cells of the immune system, however, there is a lack of evidence documenting the effect of *H. pylori* on the secretion of exosomal cytokines by these cells and, especially, on cytokines that are contained in exosomes that are released by mononuclear cells, which are predominant in chronic inflammation that is induced by persistent infection with this bacterium. On the other hand, soluble and exosomal cytokines are found in the inflammatory and tumor microenvironment (TME) and participate in intercellular communication, but what messages do the cytokines that are secreted in exosomes by mononuclear cells that are stimulated by *H. pylori* communicate? Do exosomal cytokines transmit pro- or anti-inflammatory, pro- or anti-tumor signals? Information is still lacking to answer these questions. Therefore, the aim of this study was to analyze the changes in the profile of exosomal and soluble cytokines that were secreted by PBMCs after infection with *H. pylori*, to compare the profile of exosomal and soluble cytokines. An additional purpose of this research was to bioinformatically predict the interactions that soluble and exosomal cytokines with increased levels establish with cellular proteins and, in this way, explore whether some cytokines participate in the regulation of cellular processes that are involved in carcinogenesis or cancer progression.

## 2. Results

### 2.1. H. pylori Adheres to PBMCs and Translocates CagA

**Figure 1:**
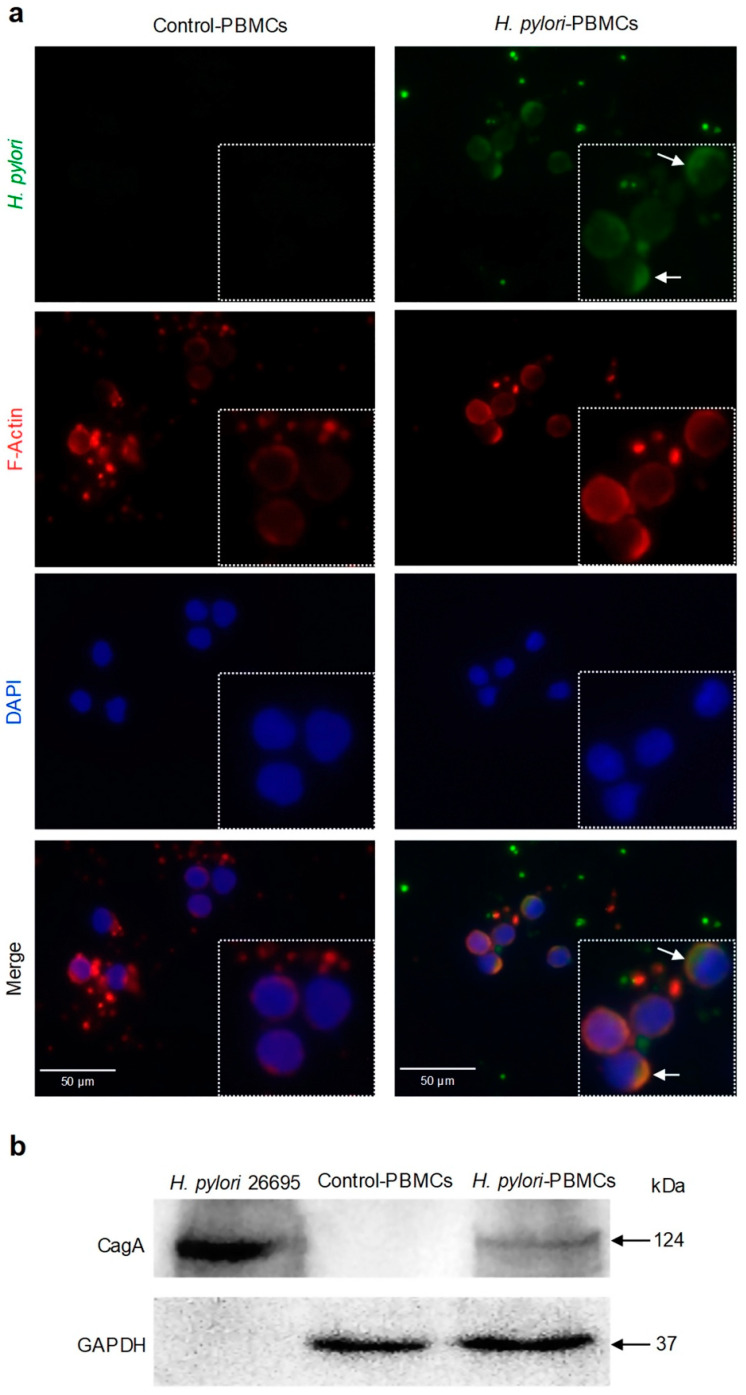
*H. pylori* adheres to PBMCs and translocates CagA. PBMCs were infected with *H. pylori* strain 26695 at a MOI of 1:50, for 24 h. (**a**) *H. pylori* infection of PBMCs was documented by immunofluorescence. White arrows indicate bacteria that were adhered to the lymphocyte membrane (green), nuclei are seen in blue, and F-actin in red. Uninfected PBMCs were used as a negative control. The images were observed under an epifluorescence microscope at 100×. (**b**) For CagA detection, 50 µg of total protein from *H. pylori*-PBMCs or Control-PBMCs were subjected to Western blot. As a positive control for CagA, 15 µg of total proteins from *H. pylori* strain 26695 were used. GAPDH was used as an internal control. All the experiments were performed in triplicate.

To assess the probability that changes in cytokines and exosomes that were secreted by PBMCs were due to *H. pylori*, through immunofluorescence and Western blot assays, it was determined whether the bacterium interacts with PBMCs. Immunofluorescence images suggest that in infected PBMCs, *H. pylori* adhered to the lymphocyte membrane (Figure 1a). This observation was confirmed with the detection of CagA among the proteins that were obtained from *H. pylori*-PBMCs, but not among the Control-PBMCs proteins. This result indicates that *H. pylori* translocated the CagA oncoprotein into the cytoplasm of lymphocytes and macrophages (Figure 1b).

### 2.2. H. pylori Infected and Uninfected PBMCs Secrete Exosomes

**Figure 2:**
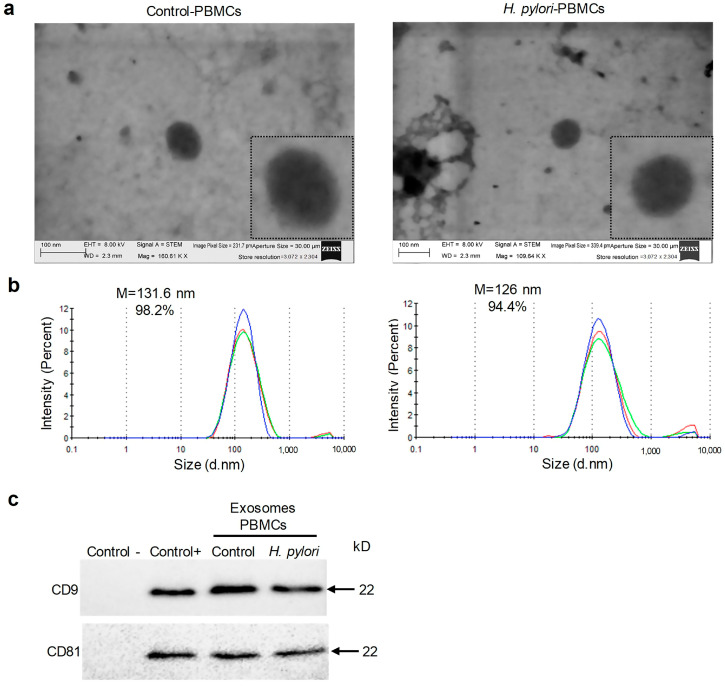
*H. pylori*-infected and -uninfected PBMCs secrete exosomes. Exosomes were isolated by ultracentrifugation. (**a**) Representative photomicrographs of exosomes that were secreted by Control-PBMCs and *H. pylori*-PBMCs at a MOI 1:100, observed in a TEM. The scale bars represent 100 nm. (**b**) Exosome particle size that was determined using the Zetasizer instrument. In the graphs, the *x* axis represents the particle size in nm and the *y* axis the percentage of particles. Each curve represents the size and percentage of exosomes. (**c**) Tetraspanins CD9 and CD81 were detected by Western blot in 30 µg of total proteins from exosomes that were secreted by Control-PBMCs and *H. pylori*-PBMCs. As a negative control, 30 μg of BSA were used and as a positive control 30 μg of proteins from exosomes that were isolated from the breast cancer cell line MDA-MB-231 were used, which secrete abundant exosomes. All experiments were performed in triplicate.

To confirm that EVs, obtained from culture supernatants of Control-PBMCs and *H. pylori*-PBMCs secreted exosomes, the vesicles were subjected to morphological analysis by transmission electron microscopy (TEM). Round vesicles were observed (Figure 2a), and particle size analysis determined that the vesicles had an average size of 70 to 150 nm (Figure 2b). Finally, Western blot confirmed the presence of tetraspanins CD9 and CD81, exosome-specific markers (Figure 2c). The results indicate that the Control-PBMCs and *H. pylori*-PBMCs secrete exosomes.

### 2.3. H. pylori Induces Soluble and Exosomal Cytokine Secretion by PBMCs

**Figure 3:**
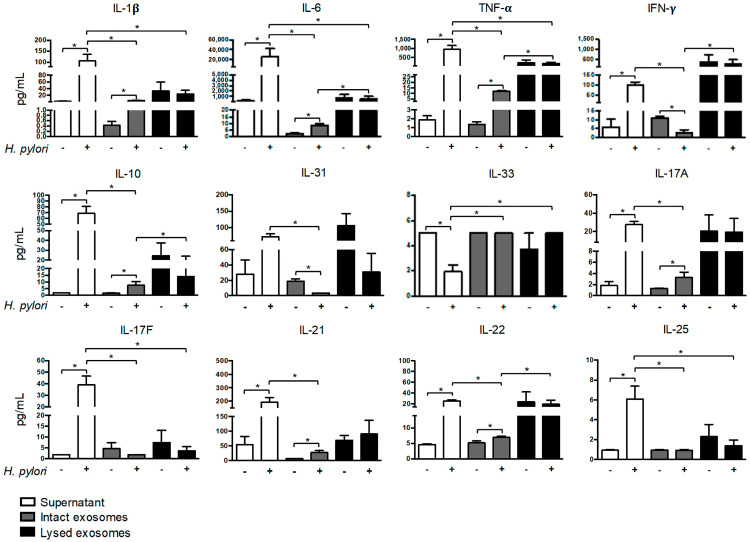
*H. pylori* induces the secretion of soluble and exosomal cytokines in PBMCs. The concentration of IL-1β, IL-6, TNF-α, IFN-γ, IL-10, IL-31, IL-33, IL-17A, IL-17F, IL-21, IL-22, and IL-25 was determined in supernatant, intact exosomes, and lysed exosomes that were secreted by the Control-PBMCs and *H. pylori*-PBMCs by a bead-based multiplex immunoassay. The graphs show the mean ± SEM. Comparison between the groups was performed using the Mann–Whitney U test, and differences were considered statistically significant when * p < 0.05. All the experiments were performed in triplicate.

To examine changes in the profile of soluble and exosomal cytokines that were secreted by *H. pylori*-PBMCs, the supernatant, intact exosomes, and lysed exosomes were subjected to a bead-based multiplex immunoassay. The cytokine concentration in pg/mL is shown in Supplementary Table S1. The concentration of IL-1β, IL-6, TNF-α, IFN-γ, IL-10, IL-17A, IL-17F, IL-21, IL-22, and IL-25 was significantly higher in the supernatant of the *H. pylori*-PBMCs than in the Control-PBMCs. In contrast, the concentration of IL-33 was lower in the supernatant of *H. pylori*-PBMCs, compared to the Control-PBMCs (Figure 3). On the other hand, *H. pylori* induced a significant increase in the concentration of IL-1β, IL-6, TNF-α, IL-10, IL-17A, IL-21, and IL-22 in intact exosomes that were secreted by PBMCs, compared to the Control-PBMCs (Figure 3). The levels of IFN-γ and IL-31 was significantly lower in the intact exosomes from *H. pylori*-PBMCs than in the Control-PBMCs (Figure 3). No significant differences were found in the concentration of IL-17A, IL-17F, IL-21, IL-22, and IL-25 in the exosomal lysate from infected and uninfected PBMCs; however, these cytokines were found at lower levels on intact exosomes from both groups of cells. *H. pylori* did not induce changes in the level of soluble or exosomal IL-4 and IL-23 (Supplementary Figure S1).

### 2.4. Cytokines Increased in Supernatant and Exosomes Secreted by H. pylori-PBMCs Are Associated with Signaling Pathways Involved in Carcinogenesis

**Figure 4:**
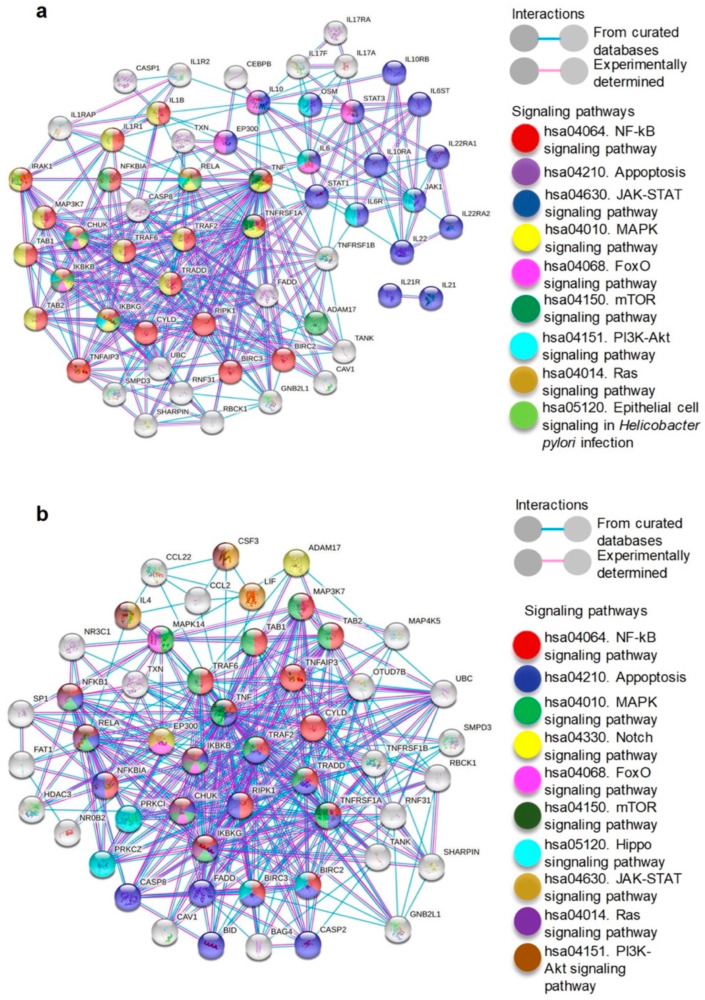
Cytokines increased in the supernatant and exosomes that were secreted by *H. pylori*-PBMCs participate in the regulation of signaling pathways that are involved in carcinogenesis. (**a**) The PPI network represents physical and functional interactions of IL-1β, IL-6, TNF-α, IL-10, IL-17A, IL-21, and IL-22 with cellular proteins that participate in signaling pathways that are related to carcinogenesis. (**b**) The PPI network constructed with TNF-α and cellular proteins. Each node represents a protein and the lines represent the interactions between the proteins (edges); pink lines denote experimentally tested interactions, and the blue lines denote the interactions with proteins from curated databases. Colored nodes represent KEGG pathways that are involved in carcinogenesis and white nodes symbolize proteins that are not involved in KEGG pathways. All interactions were statistically significant (*p* = 1.0 × 10^−16^).

The cytokines significantly increased in the supernatant and intact exosomes of *H. pylori*-PBMCs (IL-1β, IL-6, TNF-α, IL-10, IL-17A, IL-21, and IL-22), were subjected to PPI network analysis to predict their interaction with cellular proteins. The PPI network consists of 57 nodes and 334 edges, and the interactions were statistically significant (*p* = 1.0 × 10^−16^). TNF-α occupied the first position in the number of interactions (34), followed by IL-10 and IL-6 with 12 interactions (Figure 4a). Based on the results of the KEGG pathway analysis, the highest proportion of cytokine-interacting proteins participate in the NF-κB, mitogen-activated protein kinase (MAPK), phosphatidylinositol 3-kinase (PI3K)-threonine kinase (Akt), Janus kinase (Jak)-signal transducer and activator of transcription (STAT), forkhead box (FoxO), and mechanistic target of rapamycin (mTOR) signaling pathways that are related to carcinogenesis, cancer maintenance, and progression. We constructed a PPI network with TNF-α as input and generated an interactome of 51 nodes, 365 edges, and statistically significant interactions (*p* = 1.0 × 10^−16^) (Figure 4b).

TNF-α interacts with the proteins that are involved in the NF-κB, MAPK, mTOR, and apoptosis signaling pathways. These results indicate that TNF-α is the cytokine that has the most physical and functional interactions with cellular proteins.

## 3. Discussion

*H. pylori* infection induces inflammation of the gastric mucosa, which progresses to chronic gastritis, however, only a smaller proportion of individuals develop gastric cancer. The host response to the infection, the virulence factors of the bacterium, and the gastric microenvironment determine the course of the disease [22]. *H. pylori* significantly modifies the gastric microenvironment by stimulating the synthesis of chemokines, metalloproteinases, and cytokines, that are released by infiltrating cells, such as monocytes, macrophages, neutrophils, T-cells, regulatory T-cells (Treg), and natural killer (NK) cells, eventually promoting chronic gastritis and gastric carcinogenesis [3]. However, the mechanisms by which *H. pylori* promotes gastric carcinogenesis through inflammatory mediators such as cytokines or exosomes that are produced by immune cells in the gastric microenvironment are unclear. The main results of this study are, (a) *H. pylori* adheres to the membrane of lymphocytes and CagA oncoprotein enters lymphocytes and macrophages; (b) PBMCs secrete exosomes containing cytokines independently of infection status; (c) in PBMCs, *H. pylori* induces a significant increase in the secretion of soluble IL-1β, IL-6, TNF-α, IFN-γ, IL-10, IL-17A, IL-17F, IL-21, IL-22, and IL-25; (d) in PBMCs, *H. pylori* promotes increased secretion of IL-1β, IL-6, TNF-α, IL-10, IL-17A, IL-21, and IL-22 on intact exosomes; (e) concentration of IL-1β, IL-6, TNF-α, IL-10, IL-17A, IL-21, and IL-22, is significantly increased in supernatant and intact exosomes; and (f) increased cytokines in supernatant and exosomes that are secreted by *H. pylori*-PBMCs participate in the regulation of signaling pathways that are involved in inflammation, carcinogenesis or cancer progression.

With the aim of documenting whether the changes in the profile of soluble (in supernatant) and exosomal cytokines that are secreted by PBMCs are induced by *H. pylori*, it was verified whether the bacterium directly interacts with PBMCs. The results indicate that *H. pylori* strain 26695 adheres to the membrane of lymphocytes, the most abundant PBMC (approximately 70% T lymphocytes and 15% B lymphocytes) [23]. The adhesion of *H. pylori* to lymphocytes may be mediated by the interaction between the bacterium’s own molecules and molecules on the cell surface. *H. pylori* strain 26695, that was used in this research, contains LacdiNAc domain-binding adhesins (LabA), lipoproteins A and B (AlpA/AlpB), sialic acid-binding adhesin (SabA), blood group antigen-binding adhesin (BabA), and lipopolysaccharide (LPS), which are recognized by mucin 5AC (MUC5AC), laminin, Lex sialic acid (Le^x^), Lewis b antigen (Le^b^), and Toll like-receptor 4 (TLR4), respectively, which are all present in PBMCs [3,24,25,26,27]. On the other hand, for the first time it is reported that the CagA oncoprotein of *H. pylori* enters the cytoplasm of PBMCs. It is likely that *H. Pylori* has translocated CagA to the cell cytoplasm through its T4SS, which mediates the binding of the bacterium to T lymphocytes, B lymphocytes, and macrophages through the CagL protein, which contains an RGD (arginine-glycine-aspartic acid) motif [28,29,30]. CagL, encoded on the cag pathogenicity island (*cagPAI*), is a specialized adhesin that binds to the α5β1 integrin, which is present on the membrane of PBMCs [31]. The α5β1 integrin also interacts with other T4SS components, including CagY and CagI, which they contribute to stabilizing the T4SS-cell interaction and can facilitate CagA translocation [32,33,34]. The adhesion of *H. pylori* to the lymphocyte membrane and the presence of CagA within the PBMCs suggest that the bacterium induces functional changes in the infected cells, including the diversification of the cytokines that each cell subpopulation produces and changes in the concentration of these proteins.

Cytokines are messengers that participate in the communication between immune cells with healthy or tumor cells and with other cell types of the tumor microenvironment. In general, cytokines regulate the anti-tumor response, however, during chronic inflammation they can induce cell transformation and malignancy. The outcome of the interaction between the secreted cytokines in a given microenvironment depends on the balance between the pro- and anti-inflammatory cytokines, on the relative concentration of each cytokine, on the relative abundance of cytokine receptors, and the activation state of th surrounding cells. Changes in the profile of soluble cytokines that are secreted by *H. pylori*-infected PBMCs were characterized by a significant increase in the secretion of IL-1β, IL-6, TNF-α, IFN-γ, IL-10, IL-17A, IL-17F, IL-21, IL-22, and IL-25, compared to uninfected PBMCs. It has been reported that in dendritic cells, *H. pylori* induces the expression of TNF-α and that it favors the proliferation of T-cells [2]. In human macrophages, the hypothetical protein 1173 (HP1173) from *H. pylori* strain 26695 stimulates the MAPK signaling pathways, leading to the activation of NF-κB and activator protein-1 (AP-1), and thus to the secretion of TNF-α, chemokine C-X-C motif chemokine ligand 8 (CXCL8) and IL-1β [35]. In a murine model, it was found that the TNF-α-inducing protein (Tip α) of *H. pylori* activates the NF-κB signaling pathway, and contributes to the pro-inflammatory response, gastritis development, hyperplasia, and gastric cancer [36]. Moreover, *H. pylori* CagA reduces the secretion of IL-12p40, and increases the expression of IL-10, which shows pro- and anti-inflammatory effects, promoted by this oncoprotein [37]. In mice that were treated with the recombinant proteins rUreB, rCagA, or pcDNA3.1(+)-ureA plasmid from *H. pylori*, it was found that these molecules increase the secretion of IFN-γ, IL-17A, and TGF-β1 with a concomitant IL-4 diminished production and that CD4+ T-cells preferentially differentiated into Th1 and Th17 lymphocyte subpopulations; these events are characteristic of gastric inflammation [38,39,40]. In patients with non-ulcer dyspepsia, gastritis or peptic ulcers that were infected by *H. pylori*, a high secretion of IL-17, IFN-γ, IL-23, IL-6, IL-10, TNF-α, IL-21, IL-8, and IL-37 was reported [41,42,43,44,45,46,47]. In contrast, TNF-α is decreased in *H. pylori*-infected gastric cancer patients, and it has been proposed that this cytokine is involved in early stages of gastric carcinogenesis [48]. The results of this investigation suggest that *H. pylori* induces diversification and an increase in the concentration of soluble cytokines. Cytokines diversify and reinforce the effect of the bacterium on epithelial cells and on all cells in the inflammatory microenvironment, thus contributing to malignant transformation and regulation of cancer hallmarks.

Although cytokines are soluble factors, they can also reach the extracellular space through exosomes [11]. *H. pylori* induces increased exosome secretion in infected patients, human macrophages, normal gastric epithelial cells, and tumor gastric epithelial cells [17,18,19,20,21,49]. In this study, it is shown that PBMCs that were infected and uninfected with *H. pylori* secrete exosomes, however, the methods that were used make it impossible to demonstrate whether there are differences in the number of exosomes that are secreted. PBMCs including NK cells, dendritic cells, macrophages, mast cells, B lymphocytes, and T lymphocytes can secrete exosomes [50,51,52,53,54]. Previous studies show that exosomes are related to the development of gastrointestinal diseases such as chronic gastritis and gastric cancer, and also to the progression to extra-gastrointestinal diseases, including atherosclerosis [17,18,19,20,21,49,55]. On the other hand, cytokines can be released into the extracellular environment as part of the exosome cargo or as part of the exosomal membrane [11]. To our knowledge, this is the first study to report increased concentrations of IL-1β, IL-6, TNF-α, IL-10, IL-17A, IL-21, and IL-22 in intact exosomes that are secreted by PBMCs that are infected with *H. pylori* compared to PBMCs without infection. These data indicate that *H. pylori* stimulates the secretion of these cytokines in the exosomal membrane, which provides new insights into the mechanisms that are activated by the bacterium to promote intercellular communication in the microenvironment and contribute to acute or chronic inflammation, gastric carcinogenesis, or the development of extragastrointestinal diseases.

*H. pylori* induces the expression of pro-inflammatory cytokines, including IL-1β, a cytokine that promotes gastric carcinogenesis that is associated with inflammation [56]. However, it is unknown whether exosomes that are secreted by PBMCs that are infected with the bacterium transport cytokines that regulate inflammation and promote gastric carcinogenesis. In this study we found that the concentration of IL-1β, IL-6, TNF-α, IL-10, IL-17A, IL-21, and IL-22, is significantly increased in soluble form and in intact exosomes that are secreted by PBMCs that are infected with *H. pylori vacA s1m1/cagA+*. In order to explore whether this cytokine profile regulates processes that are involved in carcinogenesis, we generated a PPI interaction network between cytokines and cellular proteins. In the PPI network, we found that IL-1β, IL-6, TNF-α, IL-10, IL-17A, IL-21, and IL-22 interact directly and indirectly with proteins that participate in the NF-κB, MAPK, PI3K-Akt, mTOR, FoxO, and Jak-STAT signaling pathways. The largest number of these pathways contribute to the regulation of proliferation, differentiation, growth, apoptosis, angiogenesis, cell survival, adhesion, migration, invasion, epithelial-mesenchymal transition, resistance to oxidative stress, metabolism, autophagy, inflammation, and metastasis processes that contribute to carcinogenesis or tumor progression [57,58,59,60,61,62,63,64]. Additionally, we found that TNF-α interacts with proteins that are involved in the NF-κB, MAPK, mTOR, and apoptosis signaling pathways. TNF-α can act as an endogenous tumor promoter of inflammation, proliferation, cell transformation, survival, angiogenesis, and ultimately gastric tumor carcinogenesis or progression [5,65,66]. Evidence suggests that soluble and exosomal TNF-α may play an important role in gastric carcinogenesis, extragastrointestinal inflammation, and disease generation outside the digestive tract. In this context, chronic inflammation that is induced by *H. pylori* is one of the etiological factors of mucosa-associated lymphoid tissue (MALT) lymphoma [67,68,69]. This type of cancer affects the stomach, the lung, the ocular adnexa, the thyroid, and, to a lesser extent, the small intestine. Activated immune cells are recruited into the lymphoma and stimulate cancer cells directly by interaction with the surface receptors and/or indirectly through the secretion of soluble factors such as cytokines [69]. It has also been shown that *H. pylori* determines the type of immune response and the clinical consequences of the infection through the induction of a specific pattern of chemokines. An altered expression of chemokines may contribute to tissue damage or malignancy [67]. This background and the results that are reported here open the possibility that specific immune cells are attracted by chemokines to the site where a lymphoma is being generated and that among them are cells that are infected and/or activated by *H. pylori s1m1/cagA+* strains. On the other hand, it is likely that some chemokines travel to various tissues via exosomes and determine the type of leukocytes and immune response at the site. However, this probability has not been explored in the context of *H. pylori*-infected mucosa or gastric or extragastric diseases that are associated with this bacterium. What is well described is that cytokines act in an autocrine, paracrine, and endocrine manner; thus, these can be secreted at the site of the MALT-type lymphoma or in the *H. pylori*-infected gastric mucosa and travel through the circulation to the tumor site in soluble form or in exosomes. In this way, chemokines, cytokines, and immune cells can constitute a favorable microenvironment for the generation of lymphoma or contribute to the pathogenesis of other extragastric diseases such as cardiovascular, respiratory, metabolic, hematological, or neurological diseases [68].

## 4. Materials and Methods

### 4.1. Isolation and Culture of PBMCs

To obtain PBMCs, peripheral blood was obtained from two healthy male donors aged 23 and 28, with no history of dyspepsia, no recent viral or bacterial infection, and no antibiotic, antiviral, or anti-inflammatory treatment two weeks prior to the interview. Subjects with chronic inflammatory diseases or immunodeficiency diseases were excluded. Both donors tested negative for *H. pylori* stool antigen test (*H. pylori* AG, 04FK20, SD BIO LINE Abbott, Chicago, IL, USA). The participation of the selected subjects was voluntary, and both signed an informed consent letter in accordance with the recommendations of the declaration of Helsinki. The study was approved by the Bioethics Committee of Universidad Autónoma de Guerrero. PBMCs were obtained by density gradient centrifugation with Histopaque (1077, Sigma Aldrich, St. Louis, MO, USA). From each donor, 6 × 10^7^ PBMCs were cultured per experimental group in RPMI-1640 medium (R6504, Sigma Aldrich, St. Louis, MO, USA) that was supplemented with 5% exosome-free Fetal Bovine Serum (FBS) (Gibco, Thermo Fisher Scientific, Waltham, MA, USA). The cells in the culture were stabilized by incubation in a humid atmosphere with 5% CO_2_ at 37 °C for 24 h. All the experiments were performed in triplicate.

### 4.2. H. pylori Culture and Infection of PBMCs

*H. pylori* strain ATCC 26695 (*vacA s1m1/cagA+* genotype) was cultured on Columbia Agar containing 10% sheep blood, 10 mg/L vancomycin, 5 mg/L trimethoprim, 5 mg/L cefsulodin, 5 mg/L of amphotericin B (Oxoid, Basingstoke, UK), and polyenrichment Isovitalex (Becton, Dickinson and Company, Franklin Lakes, NJ, USA), at a pH of 6.8 to 7.0. The plates were incubated for 72 h at 37 °C, in a microaerophilic atmosphere (with O_2_ levels from 2 to 5% and 5% CO_2_). The identity of *H. pylori* was confirmed based on its colonial morphology (greyish, translucent colonies, approximately 1 mm in diameter), Gram stain (Gram-negative bacilli), and biochemical tests (urease, catalase, and oxidase-positive). From the pure culture of *H. pylori*, the bacteria were harvested and resuspended in RPMI-1640 medium (R6504, Sigma Aldrich, St. Louis, MO, USA); the suspension was adjusted to the McFarland 7.0 standard (21 × 10^8^ CFU/mL), by spectrometry [70]. These bacteria were used to infect PBMCs that were cultured for 24 h, at a multiplicity of infection (MOI) of 100:1. The cultures were incubated in a humid atmosphere with 5% CO_2_ at 37 °C for 24 h. The conditioned medium or supernatant was recovered from the cultures and stored at −70 °C until use. PBMCs without infection with *H. pylori* were designated as Control-PBMCs and PBMCs that were infected with *H. pylori* as *H. pylori*-PBMCs. All the experiments were performed in triplicate.

### 4.3. Immunofluorescence

The interaction between *H. pylori* and PBMCs was verified by immunofluorescence. A total of 1 × 10^6^ PBMCs were seeded in RPMI-1640 medium that was supplemented with 5% exosome-free FBS, in 6-well plates. The cultures were incubated at 37 °C, in a humid atmosphere with 5% CO_2_. PBMCs were infected with *H. pylori* strain 26695 at an MOI of 50:1 and incubated at 37 °C. After 24 h of infection, 50 µL of culture medium containing PBMCs and *H. pylori* were used to prepare slide smears. As a negative control, smears with 50 µL of medium with only PBMCs were used. The suspensions were left to dry for 15 min, fixed with 4% formaldehyde for 15 min, and added 100 µL of 0.1 M glycine for 20 min at room temperature, to remove the aldehyde groups. The smears were blocked with 3% BSA in phosphate-buffered saline (PBS) for 1 h at room temperature in a humid chamber, and then incubated with rabbit primary anti-*H. pylori* (PA1-73163, Thermo Fisher Scientific, Waltham, MA, USA) at a 1:100 dilution, for 4 h, at room temperature in a humid chamber. Primary antibody was detected with fluorescein-5-isothiocyanate (FITC)-labeled mouse anti-rabbit immunoglobulin G (IgG) (#sc-2359 Santa Cruz Biotechnology, Dallas, TX, USA) at a 1:100 dilution, and with rhodamine phalloidin (#R415, Thermo Fisher Scientific, Waltham, MA, USA) at a 1:100 dilution, to detect actin filaments. The smears were incubated for 2 h at room temperature in a humid chamber. To stain the nuclei, the cells were covered with Fluoroshield (40,6-diamidino-2-phenylindole, DAPI) (D1306, Thermo Fisher Scientific, Waltham, MA, USA) for 30 min and then the smears were observed under an epifluorescence microscope (Olympus model BX43, Tokyo, Japan). All the experiments were performed in triplicate.

### 4.4. Exosome Isolation and Identification

*Isolation of exosomes.* The exosomes that were secreted by Control-PBMCs and *H. pylori*-PBMCs were obtained from the culture supernatants. The medium was centrifuged at 300× *g* for 10 min at 4 °C and the supernatant was subjected to a second centrifugation at 2000× *g* at 4 °C for 10 min. Next, the supernatant was recovered and centrifuged at 10,000× *g* for 30 min; finally, the recovered medium was subjected to ultracentrifugation for 120 min at 100,000× *g*. The exosome pellet was washed with 1 mL of filtered and sterile PBS. The exosome suspension was passed through a 0.2 μm filter and centrifuged at 100,000× *g* for 70 min. The pellet was resuspended in 100 μL of PBS [71].

*Transmission electron microscopy.* To verify the morphology of exosomes that were secreted by Control-PBMCs and *H. pylori*-PBMCs, 100 μL of exosomes were fixed with 2.5% paraformaldehyde for 45 min. Next, 50 μL of exosomes were added to copper grids that were coated with Formvar/Carbon 200 Mesh Copper (FCF200-CU, Sigma Aldrich, St. Louis, MO, USA), and allowed to absorb for 20 min. The grids were allowed to dry, and the exosomes were negatively stained with uranyl acetate for 20 min. The morphology of the exosomes was analyzed in a Transmission Electron Microscope (HRSEM-AURIGA, Carl Zeiss, Jena, Germany).

*Particle size analysis.* Exosomes that were isolated from Control-PBMCs and *H. pylori*-PBMCs were diluted in nuclease-free H_2_O, in a 1:9 dilution, and analyzed using a Zetasizer instrument (Malvern Panalytical, Malvern, UK), according to the manufacturer’s protocol. The size was analyzed and the percentage of EVs was determined based on the size using the Zetasizer Nano v.3.30 software (Malvern Panalytical, Malvern, UK). All the experiments were performed in triplicate.

### 4.5. Western Blot

The total proteins of cells or exosomes were obtained with the RIPA lysis and extraction buffer. A total of 30 µg of exosomal proteins or 50 µg of cellular proteins were subjected to 10% polyacrylamide gel electrophoresis (SDS-PAGE). The proteins were transferred to a 0.2 μm nitrocellulose membrane (#1620112, Bio-Rad, Hercules, CA, USA), by electrotransfer. The membrane was blocked with 5% fat-free milk in tris-buffered saline (TBS) with Tween-20 (pH 8.0) for 2 h, under constant agitation. The membranes containing exosomal proteins were incubated overnight at 4 °C with primary anti-CD9 (D3H4P, #13403) or anti-CD81 (D3N2D, #56039) antibodies, 1:1000 dilution, both obtained from Cell Signaling (Danvers, MA, USA). The membranes with cellular proteins were incubated with primary anti-*Helicobacter pylori* CagA antibody (B818M, ab90490) 1:2500 dilution (Abcam, Cambridge, UK), or primary anti-GAPDH antibody (# sc-47724, Santa Cruz Biotechnology, Dallas, TX, USA) 1:2000 dilution. Next, the membranes were incubated for 2 h with anti-rabbit IgG secondary antibody (#7064, Cell Signaling, Danvers, MA, USA) at a 1:2000 dilution or anti-mouse IgG secondary antibody (#115-035-003, Jackson ImmunoResearch, Baltimore, PA, USA) dilution 1:2500, both coupled to horseradish peroxidase (HRP). The bands were detected by chemiluminescence (Immobilon Western Chemiluminescent HRP Substrate WBKLS0500, Burlington, MA, USA); imaging was obtained using a ChemiDoc equipment (Bio-Rad, Hercules, CA, USA), and captured with Image Lab v.5.2.1 software (Bio-Rad, Hercules, CA, USA). All the experiments were performed in triplicate.

### 4.6. Multiplex Immunoassay

The levels of cytokines were quantified in the cell culture supernatants, and in intact or lysed exosomes from Control-PBMCs and *H. pylori*-PBMCs, using the bead-based Bio-Plex Pro™ Human Th17 Cytokine Panel 15-Plex immunoassay (#171AA001M, Bio-Rad, Hercules, CA, USA). The concentration of IL-1β, TNF-α, IL-6, IFN-γ, IL- 4, IL-10, IL-31, IL-33, IL-17A, IL-17F, IL-21, IL-22, IL-23, and IL-25 cytokines were quantified following the manufacturer’s instructions. The results were analyzed with the Bio-Plex Manager v.6.0 software (Bio-Rad, Hercules, CA, USA). All the experiments were performed in triplicate.

### 4.7. Protein-Protein Interaction Network Analysis

With soluble and exosomal cytokines significantly overexpressed by the *H. pylori*-PBMCs (IL-21, TNF-α, IL-6, IL-10, IL-22, IL-1β, and IL-17A), a protein–protein interaction (PPI) network with cellular proteins was constructed. The Search Tool for Retrieval of Interacting Genes/Proteins (STRING) v.11.5 database (https://string-db.org (accessed on 14 July 2022) was used, which collects, classifies, and integrates all sources of information on protein–protein interaction that is publicly available, and includes direct (physical) and indirect (functional) interactions [72]. Cytokine reference codes were obtained from the Universal Protein (Uniprot) (https://www.uniprot.org (accessed on 13 July 2022)) and were given as input in STRING. For the prediction, only experiments and databases with a degree of confidence of 0.9 were considered. The proteins that had a direct or indirect interaction with the cytokines generated a PPI network. To identify the signaling pathways that were involved in carcinogenesis, enriched by the cytokines of interest and the proteins with which they interact, the Kyoto Encyclopedia of Genes and Genomes (KEEG) was used.

### 4.8. Statistical Analysis

The statistical analysis was carried out using the statistical software for data science (STATA) v12.0 software (College Station, TX, USA). To determine whether there are differences between groups, the Mann–Whitney U test was used. The data were plotted as the mean ± standard error (SEM) using the GraphPad Prism v.6.0 software (San Diego, CA, USA). A *p* value of <0.05 was considered statistically significant.

## 5. Conclusions

**Figure 5:**
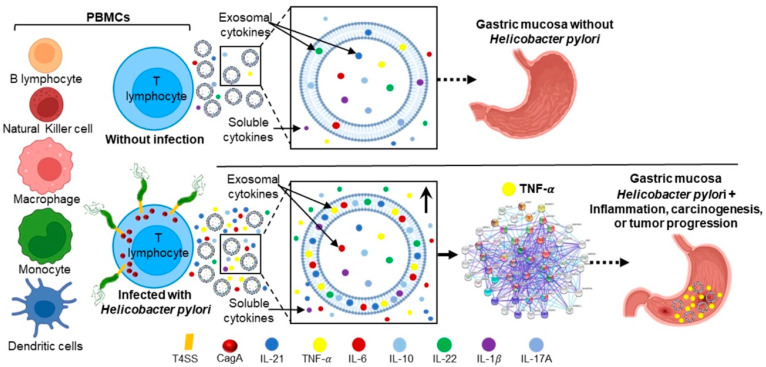
*H. pylori* promotes the secretion of exosomal and soluble cytokines that are associated with inflammation and gastric carcinogenesis. *H. pylori* adheres PBMCs and translocates CagA to the cell cytoplasm. In infected PBMCs, *H. pylori* promotes the secretion of IL-1β, IL-6, TNF-α, IL-10, IL-17A, IL-21, and IL-22. TNF-α could play an important role in the activation of signaling pathways that are associated with inflammation, gastric carcinogenesis, or tumor progression.

Overall, the results that were derived from this study demonstrate that *H. pylori* adheres to the PBMCs membrane and that CagA is internalized into PBMCs. The bacterium promotes a significant increase in the concentration of IL-1β, IL-6, TNF-α, IL-10, IL-17A, IL-21, and IL-22, in supernatant and intact exosomes. Among the soluble and exosomal cytokines that significantly increase their concentration, TNF-α is the cytokine with the highest number of interactions with proteins that regulate the NK-kB, MAPK, mTOR, and apoptosis signaling pathways, which are involved in carcinogenesis (Figure 5). These results provide information on new mechanisms of intercellular communication that are promoted by *H. pylori* to stimulate inflammation and gastric carcinogenesis. In humans, exosomes are distributed to distant organs and tissues through the circulation, so the exosomal content can contribute to the development of extragastrointestinal diseases of inflammatory origin. However, more studies are needed to clarify the specific mechanisms by which *H. pylori* induces gastric carcinogenesis, progression of cancer or extragastrointestinal diseases, through soluble and exosomal cytokines.

## Data Availability

Not applicable.

## Supplementary Materials

The supporting information can be downloaded at: https://www.mdpi.com/article/10.3390/ijms23158801/s1.
