# Supplementary material for: In Peripheral Blood Mononuclear Cells Helicobacter pylori Induces the Secretion of Soluble and Exosomal Cytokines Related to Carcinogenesis
Source: Int J Mol Sci. 2022 Aug 8;23(15):8801. doi: 10.3390/ijms23158801 (PMC9368997; doi:10.3390/ijms23158801)
Supplement: Supplementary file 1 [file ijms-23-08801-s001.zip › Table S1.pdf]

Table S1. ***H. pylori*** **promotes the secretion of exosomal and soluble cytokines associated with in-flammation and gastric**

Cytokine	Supernatant Control (pg/mL)	Supernatant H. pylori (pg/mL)	Intact exosomes Control (pg/mL)	Intact exosomes H. pylori (pg/mL)	Lysed exosomes Control (pg/mL)	Lysed exosomes H. pylori (pg/mL)
**IL-1β**	2.680±1	107.180±29	0.433±0.142	5.070±0.366	33.363±26.2	24.297±11
**IL-6**	234.170±90.2	26063.553±157	2.540±0.482	8.473±1.26	694.967±667	564.560±443
**TNF-α**	1.863±0.468	950.403±212	1.367±0.284	11.950±0.758	197.437±160	163.230±70.6
**IFN-γ**	5.763±4.7	101.603±12	10.893±0.828	2.880±1.46	376.733±366	255.147±226
**IL-4**	5.620±0	5.620±0	5.620±0	5.620±0	5.620±0	5.620±0
**IL-10**	1.960±0	68.847±11.8	1.720±0.24	7.543±2.79	24.550±12.9	14.130±9.83
**IL-31**	28.043±18.4	71.857±9.17	18.877±3.29	3.380±0	106.187±36.9	31.043±24.3
**IL-33**	5.000±0	1.950±0.504	5.000±0	5.000±0	3.713±1.29	5.000±0
**IL-17A**	1.837±0.686	27.460±3.31	1.287±0.0767	3.260±0.967	20.473±17.5	19.277±14.9
**IL-17F**	1.840±0	38.980±7.64	4.643±2.8	1.840±0	7.430±5.59	3.677±1.84
**IL-21**	53.977±28.4	191.770±35.3	6.810±0	27.290±6.7	69.080±16.4	90.407±46.7
**IL-22**	4.623±0.243	25.220±2	5.190±0.651	6.947±0.42	23.593±18.3	19.180±7.59
**IL-23**	28.350±0	53.093±15.2	28.350±0	28.350±0	28.350±0	28.350±0
**IL-25**	0.940±0.06	6.093±1.29	0.940±0.06	0.920±0.08	2.303±1.23	1.383±0.573
