# Supplementary material for: In Peripheral Blood Mononuclear Cells Helicobacter pylori Induces the Secretion of Soluble and Exosomal Cytokines Related to Carcinogenesis
Source: Int J Mol Sci. 2022 Aug 8;23(15):8801. doi: 10.3390/ijms23158801 (PMC9368997; doi:10.3390/ijms23158801)
Supplement: Supplementary file 1 [file ijms-23-08801-s001.zip › Figure S1.pdf]

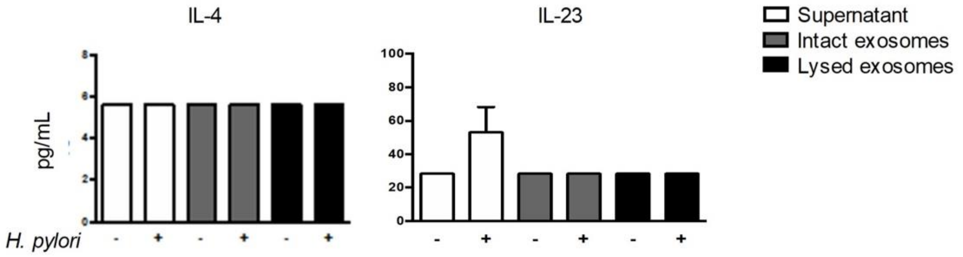

Figure S1. Cytokines secreted by Control-PBMCs and *H. pylori*-PBMCs in supernatant, and in intact or lysed exosomes. The concentration of IL-4 and IL-23 was determined in supernatant, intact exosomes and lysed exosomes secreted by Control-PBMCs or H. pylori-PBMCs. The graph represents the mean ± SEM of three independent experiments. Data were analyzed using the Mann-Whitney U test and differences were considered statistically significant when * p <0.05**.**
